# Synergistic Protective Effect of Fermented Schizandrae Fructus Pomace and Hoveniae Semen cum Fructus Extracts Mixture in the Ethanol-Induced Hepatotoxicity

**DOI:** 10.3390/antiox12081602

**Published:** 2023-08-11

**Authors:** Kyung-Hwan Jegal, Hye-Rim Park, Beom-Rak Choi, Jae-Kwang Kim, Sae-Kwang Ku

**Affiliations:** 1 Department of Korean Medical Classics, College of Korean Medicine, Daegu Haany University, Gyeongsan 38610, Republic of Korea; jegalkh@dhu.ac.kr; 2 Department of Anatomy and Histology, College of Korean Medicine, Daegu Haany University, Gyeongsan 38610, Republic of Korea; hrpark@nutracore.co.kr; 3 Nutracore Co., Ltd., Suwon 16514, Republic of Korea; brchoi@nutracore.co.kr; 4 Department of Physiology, College of Korean Medicine, Daegu Haany University, Gyeongsan 38610, Republic of Korea

**Keywords:** alcoholic liver disease, ethanol, Schizandrae Fructus, Hoveniae Semen cum Fructus, antioxidant, anti-steatosis

## Abstract

Schizandrae Fructus (SF), fruits of *Schisandra chinensis* (Turcz.) Baill. and Hoveniae Semen cum Fructus (HSCF), the dried peduncle of *Hovenia dulcis* Thunb., have long been used for alcohol detoxification in the traditional medicine of Korea and China. In the current study, we aimed to evaluate the potential synergistic hepatoprotective effect of a combination mixture (MSH) comprising fermented SF pomace (fSFP) and HSCF hot water extracts at a 1:1 (*w*:*w*) ratio against ethanol-induced liver toxicity. Subacute ethanol-mediated hepatotoxicity was induced by the oral administration of ethanol (5 g/kg) in C57BL/6J mice once daily for 14 consecutive days. One hour after each ethanol administration, MSH (50, 100, and 200 mg/kg) was also orally administered daily. MSH administration significantly reduced the serum activities of alanine aminotransferase, aspartate aminotransferase, alkaline phosphatase, and γ-glutamyl transpeptidase. Histological observation indicated that MSH administration synergistically and significantly decreased the fatty changed region of hepatic parenchyma and the formation of lipid droplet in hepatocytes. Moreover, MSH significantly attenuated the hepatic triglyceride accumulation through reducing lipogenesis genes expression and increasing fatty acid oxidation genes expression. In addition, MSH significantly inhibited protein nitrosylation and lipid peroxidation by lowering cytochrome P450 2E1 enzyme activity and restoring the glutathione level, superoxide dismutase and catalase activity in liver. Furthermore, MSH synergistically decreased the mRNA level of tumor necrosis factor-α in the hepatic tissue. These findings indicate that MSH has potential for preventing alcoholic liver disease through inhibiting hepatic steatosis, oxidative stress, and inflammation.

## 1. Introduction

Alcohol-related death was estimated to reach 3.3 million annually, accounting for 3–5% of total global deaths [1,2]. Among the various health issues associated with alcohol consumption, liver disease is the most significantly linked to heavy alcohol intake. A public health study evaluating the impact of alcohol on liver-related mortality in Europe estimated that 60–80% of deaths caused by liver disease are associated with alcohol consumption [3]. Primarily, alcohol disrupts the metabolism of liver cells, causing the accumulation of fat and acting as a hepatotoxic substance that induces oxidative stress, ultimately resulting in hepatocyte death. Alcoholic liver disease (ALD) progresses through various stages, starting with fatty liver and steatohepatitis, and it can ultimately lead to fibrosis, cirrhosis, and hepatocellular carcinoma [4]. However, the absence of reliable treatment options for severe stages of ALD has highlighted the significance of preventive measures for alcoholism and early intervention in ALD [5].

The initial stage of ALD is hepatic steatosis, which is characterized by the accumulation of fat, mainly triglycerides (TG), in hepatocytes. This pathological change is a result of the alcohol-mediated direct regulation of transcription factors associated with lipid metabolism. Acetaldehyde, a metabolite of ethanol, directly stimulates the expression of sterol regulatory element-binding protein (SREBP)-1c, which is an essential transcriptional factor that regulates the expression of genes involved in the synthesis of fatty acid [6]. On the other hand, ethanol inhibits fatty acid oxidation by suppressing the DNA binding and transcriptional activity of the peroxisome proliferator-activated receptor (PPAR) α, which governs various genes related to fatty acid transport and oxidation [7]. These imbalances between fatty acid synthesis and oxidation serves as the fundamental mechanism behind the development of fatty liver.

Oxidative stress is also a major contributing factor to the pathogenesis of ALD [4]. Most of the absorbed alcohol is metabolized by alcohol dehydrogenase to acetaldehyde, and it is further oxidized to acetic acid by aldehyde dehydrogenase in the liver. However, excessive alcohol consumption activates the 2E1 isoform of cytochrome P450 (CYP2E1) in the hepatic endoplasmic reticulum as an alternative pathway [8]. Ethanol metabolism by the CYP2E1 causes the generation of reactive oxygen species (ROS) such as hydrogen peroxide, superoxide anion, and hydroxyl radical. An improper increase in ROS in hepatocytes by ethanol induces oxidative stress and lipid peroxidation, thereby leading to the cellular damage of hepatocytes and the impairment of liver function [9]. Research on alcohol-induced liver injury using experimental animals has revealed that prolonged ethanol consumption decreases the activities of superoxide dismutase (SOD) and catalase (CAT) and the content glutathione (GSH) within liver tissue, which act as endogenous antioxidants [10,11,12]. Therefore, reducing oxidative stress through antioxidant therapies may be a potential approach to mitigate the progress of ALD.

Diverse medicinal herbs and natural products with anti-steatosis and antioxidant properties have been suggested as potent prophylactic agents for ALD [13,14]. Schizandrae Fructus (SF), fruits of *Schisandra chinensis* (Turcz.) Baill. and Hoveniae Semen cum Fructus (HSCF), dried peduncle of *Hovenia dulcis* Thunb., have been used as a remedy for alcohol-related symptoms in the traditional medicine of Korea and China [15,16]. Based on traditional usage, both herbs have been researched for the hepatoprotective effect against ethanol-induced liver injury. SF prevented the liver injury and hepatic steatosis induced by 5 weeks of ethanol administration [17]. And the identified active constituents such as lignans, triterpenoids, polysaccharides of SF have been shown to exert the anti-oxidative stress, anti-steatosis, and anti-inflammatory effects against ethanol-induced liver injury [10,18,19,20]. As for HSCF, the hepatoprotective effect against ethanol consumption has been reported by reducing oxidative stress and fatty changes of liver as well as enhancing endogenous antioxidant activity [12]. However, research about the hepatoprotective effect of SF and HSCF combination mixtures has not been conducted.

In previous research, we reported that various ratios of fermented SF pomace (fSFP) and HSCF combination mixtures (fSFP:HSCF *(w:w)*; 1:1, 1:2, 1:4, 1:6, 2:1, 4:1, 6:1, and 8:1) prevented the carbon tetrachloride (CCl_4_)-induced acute liver injury [21]. Moreover, we found that a mixture of 1:1 (*w*:*w*) fSFP and HSCF (MSH) exhibited the most potent hepatoprotective effect against CCl_4_ through enhancing the expression and the enzymatic activity of endogenous antioxidants in liver. In the present study, we further evaluated the additional hepatoprotective effect of MSH against ethanol-induced hepatotoxicity. And we also investigated the mechanisms of MSH involved in fatty acid metabolism, oxidative stress, and inflammation caused by ethanol.

## 2. Materials and Methods

### 2.1. Preparation of Silymarin, fSFP, HSCF, and MSH

Silymarin was purchased from Sigma-Aldrich (St. Louis, MO, USA). fSFP, HSCF, and MSH (1:1) extracts were supplied from Nutracore (Suwon, Republic of Korea). Briefly, 100 kg of each raw material (fSFP and HSCF) was extracted with hot water and then filtered. Finally, the resulting extracts were concentrated using an evaporator and dried using a spray drier (Figure S1). Mixed formulas (MSH) were prepared by dissolving the same amount of fSFP and HSCF in the distilled water. High-performance liquid chromatographic (HPLC) analysis determined that the MSH contained 0.6 mg/g of schizandrin (a marker compound of fSFP) and 0.17 mg/g of myricetin (a marker compound of HSCF) in MSH (Figure S2). The comprehensive procedures for the preparation of fSFP and HSCF extracts, along with the HPLC analysis of marker compounds in each extract, have been documented in the Supplementary Materials. Silymarin (–20 °C) and all test herb extracts (–4 °C) were stored in a refrigerator until usage.

### 2.2. Animal Husbandry and Experiment

A total of 80 male C57BL/6J mice (6 weeks old) were supplied from Daehan Bio Link (DBL, Eumseong, Republic of Korea), and they were acclimatized for 7 days before experiments with a 12/12 h light-cycle, 50–55% humidity, and 20–25 °C temperature-controlled conditions. Mice were allocated in eight groups (ten mice per group): vehicle, ethanol, silymarin (ethanol + 250 mg/kg of silymarin), fSFP (ethanol + 200 mg/kg of fSFP), HSCF (ethanol + 200 mg/kg of HSCF), MSH50 (ethanol + 50 mg/kg of MSH), MSH100 (ethanol + 100 mg/kg of MSH), and MSH200 (ethanol + 200 mg/kg of MSH). Ethanol (5 g/kg) was orally administered once daily for 14 consecutive days. Then, 1 h after each ethanol administration, silymarin, fSFP, HSCF, and three doses of MSH dissolved in distilled water were orally administered. The same volume of isocaloric maltose solution (as a vehicle for ethanol) or distilled water (as a vehicle for each herb extracts) was orally administered as vehicle treatment. Afterwards, 24 h after the last administration, all mice were sacrificed, and their blood and livers were collected for the further evaluation.

### 2.3. Measurements of Body and Liver Weight

Body weight was measured at 1 day before the first oral gavage, the day of first administration (day 0), and at 1, 7, 10, 13, 14 days after the first administration (total 7 times) using an electronic balance (XB320M, Precisa Instrument, Dietikon, Switzerland). Body weight gains were calculated as subtracting the body weight on the last day of administration (day 14) from the body weight on the first day of administration (day 0). Liver weight was measured immediately at sacrifice and represented as a ratio to the body weight of the individual (g/g).

### 2.4. Serum Biochemistry

First, 1 mL of venous blood was collected from the vena cava at sacrifice and centrifuged (12,500 rpm, 10 min, 4 °C) to separate serum. Activities of alanine aminotransferase (ALT), aspartate aminotransferase (AST), alkaline phosphatase (ALP), γ-glutamyl transpeptidase (GGT), and level of TG in serum were measured using an automated blood analyzer (Dri-Chem NX500i; Fuji Medical System, Tokyo, Japan).

### 2.5. Histopathological Analysis and Immunohistochemistry

After the sacrifice, the left lateral lobes of the harvested liver were fixed in 10% neutral-buffered formalin. Equal regions of fixed left lateral lobes were crossly trimmed and divided into two parts. One was embedded in paraffin and serially sectioned (3–4 μm thickness, three sections per single liver tissue) for general histopathological observation using hematoxylin and eosin or immunohistochemistry. The other part of the liver tissues was dehydrated in 30% sucrose solution, sectioned by cryostat, and stained with oil red. The representative histological profiles of individual samples were observed using a light microscope (Model Eclipse 80*i*, Nikon, Tokyo, Japan). The numbers of hepatocytes occupied with over 20% of lipid droplets in cytoplasm (cells/1000 hepatocytes), the percentages of fatty changed regions (%/mm^2^), and the mean hepatocytes diameter (μm/hepatocytes) in ten fields of single slide were calculated using an automated image analyzer (*i*Solution FL ver 9.1, IMT *i*-solution Inc., Burnaby, BC, Canada), according to the previously established method [11,12]. The average values of ten mice were represented as the results.

For the immunohistochemistry, deparaffinized sections were heated (95–100 °C) in 10 mM citrate buffer for antigen retrievals. Furthermore, sections were incubated in methanol and 0.3% hydrogen peroxide for 30 min to inhibit endogenous peroxidase activity and blocked with normal horse serum to prevent the non-specific binding of antibodies. After incubation with primary and biotinylated secondary antibodies, immunoreactive cells were visualized with an avidin–biotin complex kit (Vector Labs, Burlingame, CA, USA). The numbers of cells with more than 20% immunoreactivity, regarded as positive, in the restricted view area of the hepatic parenchyma was measured using an automated image analyzer (IMT *i*-solution Inc.), as previously described [11,12].

### 2.6. Enzyme-Linked Immunosorbent Assay (ELISA)

To measure hepatic TG contents, the right lobes of liver were homogenized with phosphate-buffered saline (PBS) using a bead homogenizer (taco™Pre, GeneReach Biotechnology, Taichung, Taiwan) and ultrasonic disruptor (KS-750, Madell Technology, Ontario, CA, USA). The TG contents in liver homogenates were measured using a commercial ELISA kit (Mybiosource, San Diego, CA, USA), as previously reported [12]. Finally, hepatic TG contents were normalized with a total protein content.

To measure the level of tumor necrosis factor (TNF)-α, liver tissues with ice-cold radioimmunoprecipitation assay buffer were centrifuged at 20,000× *g* for 15 min at 4 °C temperature. The TNF-α concentration in the resulting supernatant was quantified with a commercial ELISA kit (Mybiosource). Each of the measured values was normalized with the total protein content of the samples.

Contents of total protein in each sample were measured by the Lowry method using bovine serum albumin as the standard [22].

### 2.7. Quantitative Reverse Transcription Polymerase Chain Reaction (RT-qPCR)

The total RNA was isolated using TRIzol reagent (Invitrogen, Carlsbad, CA, USA), and cDNA was synthesized using the High-Capacity cDNA Reverse Transcription kit (Thermo Fisher Scientific, Rockford, IL, USA), according to the manufacturer’s instructions. Real-time PCR was performed using CFX96™ (Bio-Rad, Hercules, CA, USA) for the quantification of mRNA expression. Primer sequences used for the amplification of each target mRNA are listed in Table 1. For the quantitative measure of each mRNA expression, the expression in hepatic tissue of the vehicle-treated group was used as the control, and the expression of mRNA was normalized with the expression of β-actin mRNA. The relative mRNA expression of each gene was calculated using the 2^−ΔΔCt^ method [23].

### 2.8. Measurement of Liver Lipid Peroxidation

Liver lipid peroxidation was estimated in terms of malondialdehyde (MDA) concentration in hepatic homogenates. Hepatic tissues were homogenized in the ice-cold 0.01 M Tris-HCl (pH 7.4) solutions and centrifuged at 12,000× *g* for 15 min. After reaction with thiobarbituric acid (TBA), the MDA–TBA adduct was quantified by detecting absorbance at 525 nm. MDA concentration was normalized with the total protein content of each sample.

### 2.9. Measurement of Antioxidant Capacities in the Liver

Liver tissues with PBS were homogenized using a bead homogenizer (GeneReach Biotechnology) and ultrasonic disruptor (Madell Technology). After centrifugation, the resulting supernatant was collected as liver homogenates. The GSH level, SOD activity, and CAT activity were measured in hepatic homogenates as previously reported [11]. Briefly, to measure the GSH level, liver homogenates were mixed with 100 µL of 25% trichloroacetic acid and centrifuged at 4200 rpm for 40 min. After reaction with 2-nitrobenzoic acid, absorbance at 412 nm was detected. Here, 1 unit of CAT activity was defined as the amount of enzyme needed to catalyze the decomposition of 1 nM of hydrogen peroxide per minute at 25 °C and pH 7.8 conditions. And CAT activity was calculated by assessing the breakdown of hydrogen peroxide by CAT within the liver homogenate using absorbance readings at 240 nm. Superoxide radicals react with nitrotetrazolium blue (NBT) to produce formazan dye. SOD activity was measured by assessing the extent of inhibition of this reaction. One unit of SOD activity was defined as the amount of enzyme needed to inhibit NBT reduction by 50% within 1 min. Superoxide radicals were generated by xanthine and xanthine oxidase, and the degree of NBT reduction inhibition by SOD within liver homogenates was measured through absorbance readings at 560 nm. Measurement results of SOD and CAT activity were normalized with the protein content of each sample and expressed as U/mg protein.

### 2.10. Determination of Liver CYP2E1 Activity

CYP2E1 catalyzes the hydroxylation of *p*-nitrophenol to 4-nitrocatechol. Therefore, the concentration of the created 4-nitrocatechol by reaction in liver homogenates was determined by colorimetrical analysis [12,24]. Briefly, liver homogenates were centrifuged at 10,000× *g* for 15 min, and supernatants were further centrifuged (105,000× *g*, 60 min) to obtain microsomes. Absorbance at 535 nm was measured to detect the concentration of 4-nitrocatechol. The CYP2E1 activities were expressed as micromoles of 4-nitrocatechol formed per minute per mg of microsomal protein (μM/min/mg protein).

### 2.11. Statistical Analysis

All values were expressed as mean ± standard deviation. According to the homogeneity of variance, a one-way analysis of variance (ANOVA) test or Kruskal–Wallis H test was conducted. If significant difference was observed among the group, Tukey’s Honest Significant Difference test or a Mann–Whitney U test was conducted to determine the statistically significant difference in the specific pairs of groups. All statistical analyses were conducted using SPSSS 14.0 (IBM SPSS Inc., Armonk, NY, USA).

## 3. Results

### 3.1. MSH Synergistically Ameliorated Ethanol-Induced Liver Injury in Mice

To explore the hepatoprotective effects of MSH, mice were orally administered with ethanol (5 g/kg) once daily for 14 consecutive days. One hour after each ethanol administration, Silymarin (250 mg/kg), fSFP (200 mg/kg), HSCF (200 mg/kg), and three doses of MSH (50, 100, and 200 mg/kg) were administrated for 14 days. Silymarin consistently demonstrated a protective effect against animal experimental models of liver disease using CCl_4_, acetaminophen, or thioacetamide [25,26,27]. Additionally, in the experimental model of ALD, it proved to be effective via reducing ethanol-induced lipid peroxidation, TG accumulation, and TNF-α secretion in the liver [24]. In previous reports, we also confirmed its effectiveness against 14 days of daily ethanol consumption (5 g/kg) at a concentration of 250 mg/kg [11,12]. Due to these consistent and reliable effects, silymarin was selected as the positive control drug for this study.

**Figure 1:**
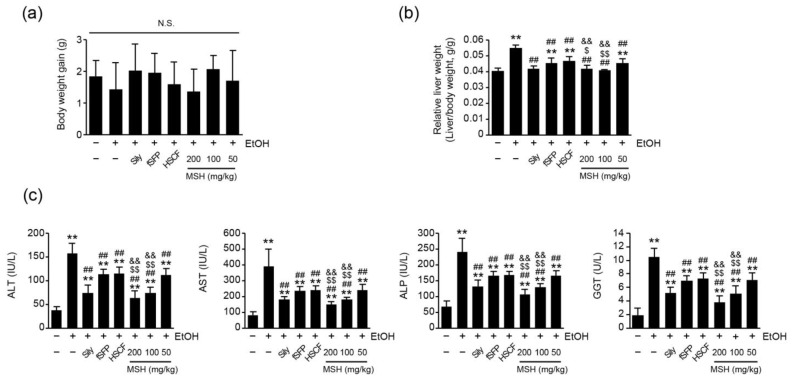
Effect of fermented Schizandrae Fructus Pomace (fSFP) and Hoveniae Semen cum Fructus (HSCF) combination mixture (MSH) on body weight gain, liver weight, and serum activities of liver enzymes in ethanol intoxicated mice. (**a**) Body weight gain was calculated by subtracting the body weight of day 0 from those of day 14. (**b**) Relative liver weight. Absolute liver weight was divided by the body weight of each individual. (**c**) Enzymatic activity of liver enzymes in serum. Activities of alanine aminotransferase (ALT), aspartate aminotransferase (AST), alkaline phosphatase (ALP), and γ-glutamyl transpeptidase (GGT) were measured in serum. All values were expressed as the mean ± standard deviation of 10 mice. Significant versus vehicle group, ** *p* < 0.01; versus EtOH group, ^##^ *p* < 0.01; versus fSFP-treated group, ^$^ *p* < 0.05, ^$$^ *p* < 0.01; versus HSCF-treated group, ^&&^ *p* < 0.01. N.S., not significant; EtOH, ethanol; Sily, silymarin.

Gains of body weight between the first (day 0) and the last day (day 14) of administration showed no statistical significance among all treatment groups (Figure 1a). As previously reported [11,12], the relative liver weight, representing absolute liver weight divided by the body weight, was significantly increased after 14 days of ethanol administration compared to the vehicle control group. However, silymarin, fSFP, HSCF, and three doses of MSH (50, 100, and 200 mg/kg) significantly reduced the elevation of relative liver weight by ethanol administration. Especially, the reduction in relative liver weight achieved through 100 and 200 mg/kg of MSH administration was significantly greater than those observed in fSFP or HSCF single administration (Figure 1b).

Next, we assessed the effect of MSH on the serum biomarkers of liver function. We found that 14 days of ethanol administration significantly increased the serum activities of ALT, AST, ALT and GGT, indicating hepatic injury. However, silymarin, fSFP, HSCF, and MSH (50–200 mg/kg) administration significantly reduced the activities of ALT, AST, ALP, and GGT in the serum. Especially, 100 and 200 mg/kg of MSH administration showed a significantly greater reductive effect on the ALT, AST, ALT, and GGT activities than the single administration of fSFP or HSCF (Figure 1c).

### 3.2. MSH Synergistically Alleviated Ethanol-Induced Hepatic Steatosis in Mice

**Figure 2:**
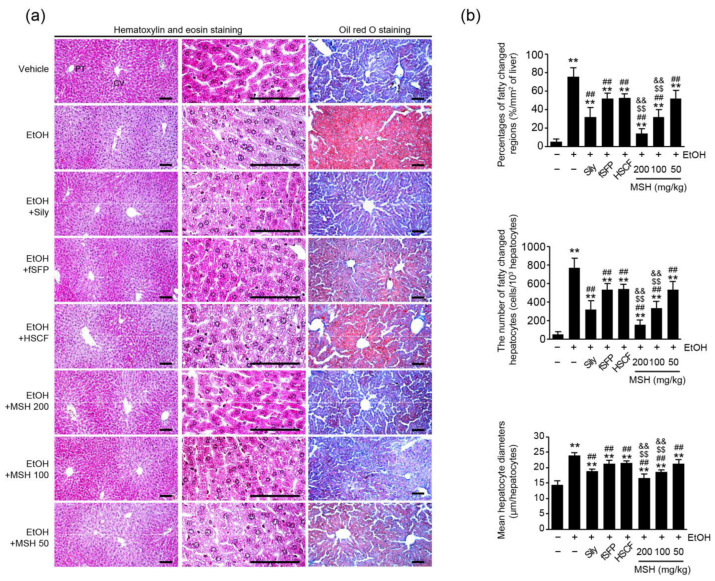
Effect of MSH on the hepatic steatosis in ethanol-intoxicated mice. (**a**) Representative profile images of hematoxylin and eosin-stained (**left**) or oil red O-stained (**right**) liver tissues for histopathological observation. In the oil red O staining, nuclei were counterstained with hematoxylin solution. Scale bars indicate 200 μm. (**b**) Histopathological analysis. Percentage of fatty changed regions (**upper**), the number of fatty changed hepatocytes (**middle**), and mean hepatocyte diameter (**lower**) were observed using an automated image analyzer. Significant versus vehicle group, ** *p* < 0.01; versus EtOH group, ^##^ *p* < 0.01; versus fSFP-treated group, ^$$^ *p* < 0.01; versus HSCF-treated group, ^&&^ *p* < 0.01. CT, central vein; PT, portal triad; EtOH, ethanol; Sily, silymarin.

Hepatic steatosis is a major pathological feature of alcohol mediated-liver disease [4]. Therefore, we assessed fatty changes of hepatic tissues by histological observation using hematoxylin–eosin and oil red O stain (Figure 2a). Histomorphological analysis revealed that the percentage of fatty changed regions, the number of fatty changed hepatocytes, and the mean hepatocyte diameters were significantly increased after 14 days of ethanol administration, indicating fatty changes of the liver. However, silymarin, fSFP, HSCF, and MSH (50–200 mg/kg) administration significantly reduced hepatocytes with lipid droplets, fatty regions of hepatic parenchyma, and means of hepatocytes diameter. Especially, the reductions of hepatic fatty changes achieved through 100 and 200 mg/kg of MSH administration were significantly greater than those observed after the single administration of either fSFP or MSH (Figure 2b).

### 3.3. MSH Alleviated Ethanol-Induced Hepatic TG Accumulation via Modulating Fatty Acid Metabolism

**Figure 3:**
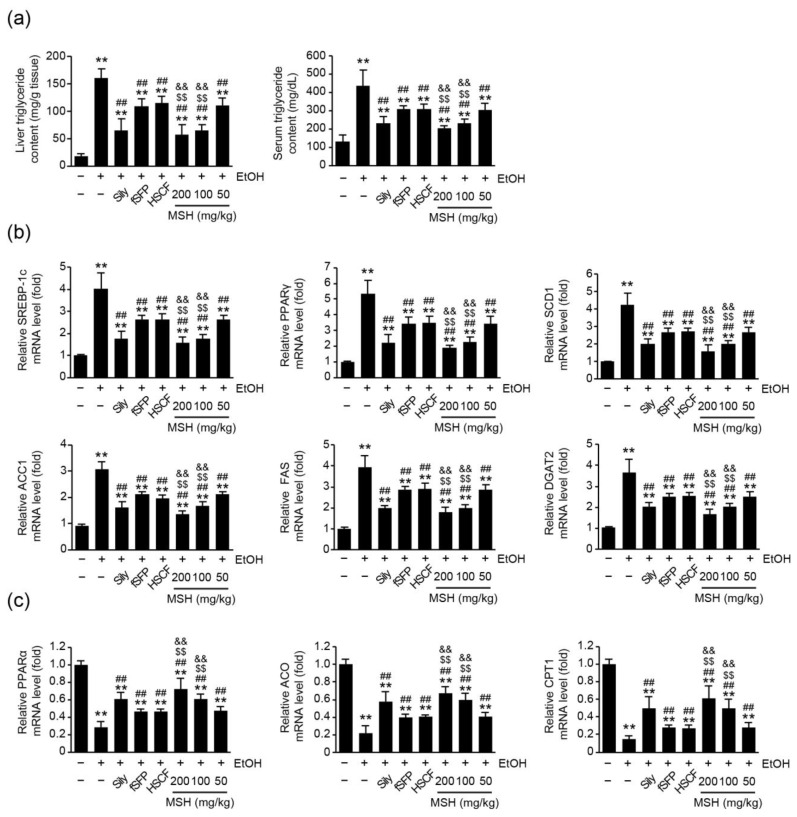
Effect of MSH on triglycerides synthesis and fatty acid metabolism in the ethanol-intoxicated mice liver. (**a**) Triglyceride level in hepatic tissue (**left**) and serum (**right**). (**b**) Expression of genes related to fatty acid and triglyceride synthesis. Relative mRNA levels of SREBP-1c, PPARγ, SCD1, ACC1, FAS, and DGAT2 were measured. (**c**) Expression of genes related to fatty acid oxidation. Relative mRNA levels of PPARα, ACO and CPT1 were measured. mRNA level of each gene was measured using RT-qPCR and normalized with β-actin gene expression. Significant versus vehicle group, ** *p* < 0.01; versus EtOH group, ^##^ *p* < 0.01; versus fSFP-treated group, ^$$^ *p* < 0.01; versus HSCF-treated group, ^&&^ *p* < 0.01. EtOH, ethanol; Sily, silymarin.

Next, we investigated whether MSH ameliorates ethanol-induced lipid accumulation in hepatic tissue through modulating fatty acid metabolism. First, hepatic and serum TG levels were assessed to elucidate the effect of MSH on the TG synthesis. We found that 14 days of ethanol administration significantly increased the levels of hepatic and serum TG. However, silymarin, fSFP, HSCF, and three doses of MSH (50–200 mg/kg) significantly decreased the levels of hepatic and serum TG (Figure 3a). Furthermore, the mRNA levels of genes related to fatty acid metabolism were assessed using RT-qPCR to elucidate the modulative effect of MSH on the fatty acid metabolism. As is known [11,28], 14 days of ethanol administration significantly increased the relative mRNA expressions of genes including SREBP-1c, PPARγ, stearoyl-CoA desaturase 1 (SCD1), acetyl-CoA carboxylase 1 (ACC1), fatty acid synthase (FAS), and diglyceride acyltransferase 2 (DGAT2), which are involved in fatty acid and TG synthesis. Conversely, mRNA expressions of PPARα, acyl-CoA oxidase (ACO), and carnitine palmitoyltransferase 1 (CPT1), related to fatty acid oxidation, were significantly inhibited by ethanol administration. However, silymarin, fSFP, HSCF, and MSH (50–200 mg/kg) significantly restored the ethanol-induced changes in the mRNA expression of genes responsible for fatty acid metabolism (Figure 3b,c). Moreover, the restorative effect of MSH (100 and 200 mg/kg) on the elevation of lipogenic genes expression and the reduction in lipolytic genes expression by ethanol was significantly greater than those after the single administration of fSFP or HSCF (Figure 3b,c).

### 3.4. MSH Inhibited Ethanol-Mediated Oxidative Stress and Inflammation in Mice

**Figure 4:**
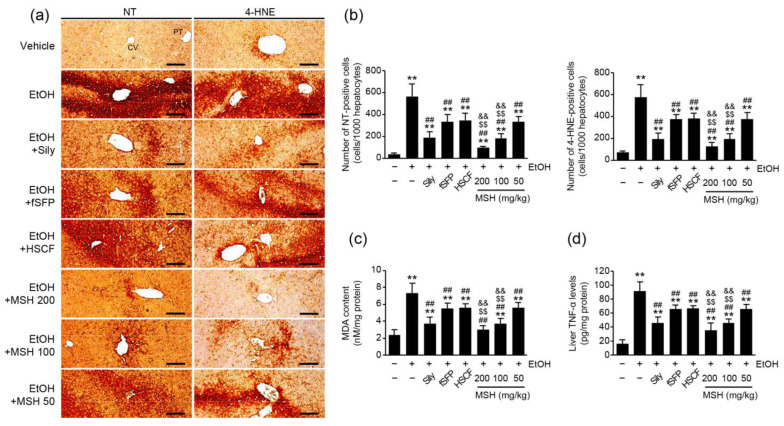
Effect of MSH on the ethanol-induced oxidative stress and inflammation. (**a**) Representative profiles of immunohistochemistry using anti-NT and 4-HNE antibodies. Scale bars indicate 200 μm. (**b**) The numbers of NT- (**left**) and 4-HNE- (**right**) positive cells. Cells showing more than 20% of immunoreactivity were counted. (**c**) MDA content and (**d**) tumor necrosis factor (TNF)-α in the hepatic tissue were quantified using liver homogenates. Significant versus vehicle group, ** *p* < 0.01; versus EtOH group, ^##^ *p* < 0.01; versus fSFP-treated group, ^$$^ *p* < 0.01; versus HSCF-treated group, ^&&^ *p* < 0.01. CT, central vein; PT, portal triad; EtOH, ethanol; Sily, silymarin; NT, nitrotyrosine; 4-HNE, 4-hydroxynonenal; MDA, malondialdehyde.

Along with steatosis, oxidative stress and inflammation are the main etiologies of ethanol-induced hepatotoxicity [4]. To elucidate the effect of MSH on the ethanol-induced oxidative stress in liver, we measured the expressions of nitrotyrosine (NT; a nitrosative stress marker) and 4-hydroxynonenal (4-HNE; a lipid peroxidation marker) using immunohistochemistry (Figure 4a). Results showed that ethanol administration significantly increased the numbers of NT- and 4-HNE immunopositive cells in the hepatic parenchyma. However, administration of silymarin, fSFP, HSCF, and MSH (50–200 mg/kg) significantly decreased the numbers of NT- and 4-HNE positive cells (Figure 4b). Moreover, the content of MDA, another product of lipid peroxidation, in the liver homogenates was elevated by ethanol administration. However, it was significantly decreased by silymarin, fSFP, HSCF and MSH (50–200 mg/kg) administration (Figure 4c). In addition, the ethanol-mediated elevation of TNF-α, which known as a pro-inflammatory cytokine, in hepatic homogenates was significantly inhibited by silymarin, fSFP, HSCF, and MSH (50–200 mg/kg) administrations (Figure 4d). Especially, the reduction in these oxidative stress (NT, 4-HNE, and MDA) and inflammation (TNF-α) markers achieved through 100 and 200 mg/kg of MSH administration was significantly greater than those observed in either fSFP or HSCF single administration.

### 3.5. MSH Enhanced the Endogenous Antioxidant Capacity and Lowered the CYP2E1 Activity in Liver

**Figure 5:**
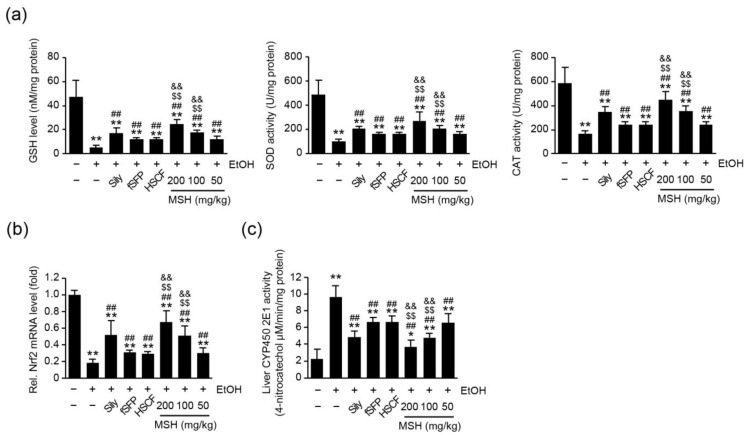
Effect of MSH on the antioxidant system and CYP2E1 activity in ethanol-intoxicated mice liver. (**a**) Endogenous antioxidant capacities. The glutathione (GSH) level (**left**), superoxide dismutase (SOD) (**middle**), and catalase (CAT) (**right**) activities were measured in liver homogenates. (**b**) Relative level of Nrf2 mRNA. Nrf2 mRNA level was measured using RT-qPCR and normalized with β-actin mRNA expression. (**c**) CYP450 2E1 activity. Significant versus vehicle group, * *p* < 0.05, ** *p* < 0.01; versus EtOH group, ^##^ *p* < 0.01; versus fSFP-treated group, ^$$^ *p* < 0.01; versus HSCF-treated group, ^&&^ *p* < 0.01. EtOH, ethanol; Sily, silymarin.

Numerous researchers have reported that ethanol consumption in the experimental animal model induced the depletion of endogenous antioxidant capacities [11,12,24,29]. Therefore, we measured the level of GSH and activities of SOD and CAT in the hepatic tissue to investigate whether the hepatoprotective effect of MSH on ethanol-induced toxicity is exerted through restoring endogenous antioxidants. Ethanol administration significantly reduced the level of GSH as well as activities of SOD and CAT in liver. However, silymarin, fSFP, HSCF, and MSH (50–200 mg/kg) administration significantly restored the level of GSH as well as activities of SOD and CAT. Moreover, the extent of elevations in the GSH level, SOD and CAT activities by MSH (100 and 200 mg/kg) was significantly greater than those by fSFP or HSCF single administration (Figure 5a). In addition, MSH (50–200 mg/kg) administration significantly and dose-dependently increased the mRNA level of nuclear factor erythroid 2-related factor-2 (Nrf2), which is known as a master transcriptional regulator of antioxidants genes. And its extent of increase was significantly greater than those observed in fSFP or HSCF single administration (Figure 5b).

CYP2E1 is considered as one of the major contributors on ethanol-mediated ROS generation in liver [4,30,31]. We assessed the activity of CYP2E1 in a liver homogenate to investigate whether MSH contributes to the reduction in ethanol-induced ROS via the regulating activity of CYP2E1. As a result, MSH (50–200 mg/kg) administration significantly reduced the activity of CYP2E1 (Figure 5c). These results suggested that MSH lowered the oxidative stress in the hepatic tissue via enhancing antioxidant capacities and lowering CYP2E1 activities.

## 4. Discussion

Despite the profound impact of alcohol abuse on public health, few therapeutic approaches have been approved for the treatment of patients with ALD [5]. Therefore, numerous studies were conducted to find the medicinal herbs and natural compounds possessing hepatoprotective effects for the prevention of ALD [13,14]. Especially, *S. chinensis* and *H. dulcis* have been used as a key ingredient herb for treating alcohol detoxification in Korean and Chinese traditional medicine [15,16]. The ethnopharmacological evidence has prompted research about the hepatoprotective effect of SF and HSCF against ALD [12,17,32]. In previous study, we evaluated the hepatoprotective effect fSFP and HSCF (*w*:*w*; 1:1, 1:2, 1:4, 1:6, 2:1, 4:1, 6:1 and 8:1) mixtures at different ratios to develop a novel functional food for preventing liver disease [21]. Among the various ratios of mixtures, we concluded that the 1:1 mixture of fSFP and HSCF (MSH) showed the most potent hepatoprotective effect in CCl_4_-induced liver injury mice via lowering oxidative stress and inflammatory response. And this mixture showed a greater hepatoprotective effect than fSFP or HSCF single administration. Therefore, in the current study, we aimed to investigate the synergistic protective effect of a fSFP and HSCF 1:1 mixture (MSH) on ethanol-induced liver toxicity.

One of the early pathological responses to prolonged alcohol consumption is the fat accumulation in hepatocytes, which is a condition called hepatic steatosis [28]. With fatty changes of liver, ethanol-induced oxidative stress and inflammatory response intensify the damage of liver [4]. In the present study, histomorphometrical observation showed the significant fatty changes of hepatic parenchyma and hepatocytes after 14 days of ethanol feeding. Moreover, ALT, AST, ALP, and GGT activities, as serum markers of liver damage, were elevated, as previous research showed [11]. However, ethanol-induced hepatic steatosis was significantly and dose-dependently reduced by MSH administration (50–200 mg/kg), showing decreases in the percentage of fatty changed regions, the number of fatty changed hepatocytes, and the mean of hepatocyte diameters (Figure 2). And activities of ALT, AST, ALP, and GGT in the serum were also significantly reduced by MSH administration (50–200 mg/kg) (Figure 1c). In addition, a significantly greater restorative effect of MSH on fatty changes of liver and serum markers, compared to fSFP or HSCF single administration, suggested the synergistic protective effect of MSH against ethanol-induced steatosis and liver damage.

An imbalance between lipid synthesis and fatty acid oxidation results in the excessive accumulation of fat in the liver, playing a crucial role in the progression of ALD [28]. In the current study, we found that ethanol increased the hepatic TG accumulation and mRNA expression of SREBP-1c, PPARγ, and its target genes (Figure 3a,b). SREBP-1c, the predominant isoform of SREBP in liver, acts as a transcriptional factor regulating the expression of genes related to fatty acid synthesis, such as SCD1, ACC1, and FAS. It has been reported that ethanol treatment promoted fatty acid synthesis via enhancing the transcriptional activity of SREBP-1 [6]. PPARγ, a type II nuclear receptor, also controls the expression of lipogenic genes such as DGAT2, which is involved in TG synthesis. The hepatic knockdown of PPARγ-attenuated chronic alcohol feeding induced hepatic steatosis and injury, inhibiting the expression of SREBP-1c [33]. In addition to elevated lipogenesis, reduced fatty acid oxidation also contributes to the development of fatty liver by ethanol consumption. In our experiment, 14 days of ethanol administration decreased the mRNA expression of PPARα, CPT1, and ACO, which is involved in fatty acid oxidation. As a subtype of the PPAR superfamily, PPARα regulates the expression of genes involved in the transport and mitochondrial oxidation of fatty acid [34]. Research showed that ethanol feeding impaired the expression of PPARα response genes [35]. Conversely, agonists of PPARα showed a therapeutic effect against the ethanol-induced liver injury via modulating lipid metabolism [35,36,37]. In our results, MSH administration restored the gene expression of not only fatty acid synthesis but also fatty acid oxidation in hepatic tissue (Figure 3). These results suggested that MSH inhibited ethanol-induced TG accumulation in the liver by modulating lipid metabolism.

Ethanol is mainly oxidized by alcohol dehydrogenase to acetaldehyde in hepatocytes. However, when alcohol dehydrogenase becomes saturated by excessive ethanol, CYP2E1-mediated-alcohol metabolism is activated as an alternative pathway [8]. The chronic consumption of alcohol increases the expression and activity of CYP2E1, thereby promoting the generation of ROS during the metabolization of ethanol [28,38]. Numerous research studies have reported that CYP2E1 expressions and activity are responsible for the severity of ethanol-mediated liver injury. Studies using transgenic mice showed that CYP2E1 overexpression aggravates ethanol-induced hepatic injury [30,31]. On the other hand, genetic deletion and the pharmacological inhibition of CYP2E1 attenuated alcohol-induced hepatic damage [39,40]. Taken together, the evidence provides support for CYP2E1 being an important factor in the progress of ALD. Therefore, the development of drugs targeting CYP2E1 has been attempted for the treatment of early stages of ALD [38]. The inhibitory effects of *S. chinensis* and *H. dulcis* on ethanol-induced CYP2E1 activation have also been studied. The total lignans of *S. chinensis* significantly reduced the protein and mRNA level of CYP2E1 in alcohol-induced liver injury rats [32]. In addition, we reported that HSCF administration significantly inhibited CYP2E1 enzymatic activities in ethanol-intoxicated mice [12]. In the present study, the elevated CYP2E1 activity by ethanol administration was significantly reduced by MSH administration. Especially, 100 and 200 mg/kg of MSH administration exhibited the greater inhibitory effect on CYP2E1 activity than either fSFP or HSCF single administration (Figure 5c). Eventually, the generation of ROS by chronic ethanol exposure causes the lipid peroxidation. The reaction between generated radical and hepatic lipid led to the formation of reactive aldehyde substances like 4-HNE and MDA. Because of their high reactivity, 4-HNE and MDA can affect the structure and function of major biomolecules such as proteins and DNA [41]. One study reported that patients with ALD showed increases in the MDA and 4-HNE level in the serum [42,43]. In the present study, MSH administration significantly reduced the elevated number of 4-HNE-positive cells in hepatic parenchyma and content of MDA in hepatic homogenates by ethanol administration (Figure 4). These results suggested that MSH attenuated ethanol-induced oxidative stress in liver by inhibiting CYP2E1-mediated ROS generation and lipid peroxidation.

The abnormal expression of pro-inflammatory cytokines is another major feature of ALD [44]. Especially, chronic alcohol consumption increased TNF-α production in the hepatic tissue, contributing to liver injury [45,46]. Monocytes and Kupffer cells become more sensitive to lipopolysaccharide after continuous alcohol consumption, leading to further TNF-α production [47,48]. Elevated TNF-α secretion is considered a major determinant of susceptibility to ethanol-induced liver toxicity. TNF-α type I receptor-deficient mice fed long-term ethanol did not develop liver injury [49]. Moreover, the neutralization of TNF-α by specific antibodies attenuated hepatic inflammation and necrosis in the ethanol-fed rats [46]. In the current study, subacute ethanol administration elevated the hepatic TNF-α production in hepatic tissue (Figure 4d). However, MSH (50–200 mg/kg) administration significantly reduced the hepatic TNF-α expression induced by ethanol, suggesting that MSH attenuated the severity of ethanol-induced liver injury via modulating the inflammatory response.

Normally, SOD and CAT, as enzymatic antioxidants, contribute to the redox homeostasis of liver tissue by scavenging superoxide and hydrogen peroxide respectively. As a non-enzymatic antioxidant, GSH is involved in the detoxification of xenobiotics and neutralizing harmful free radicals [50]. One study using transgenic mice showed that SOD1 knockout promoted the ethanol-induced oxidative stress and liver injury [51]. In addition, the depletion of mitochondrial GSH increased the susceptibility of hepatocytes to TNF-α-mediated cell death in ethanol-fed rat hepatocytes [52]. Although hepatocytes are equipped with these antioxidant systems to defend against oxidative stress, studies using laboratory animals revealed that chronic alcohol exposure reduces the activity of SOD, CAT, and depleted GSH content, thereby causing oxidative stress-mediated liver injury [12,29]. Consistent with these facts, we found that ethanol administration significantly reduced the SOD, CAT activities, and GSH level in the hepatic homogenates. However, MSH administration significantly restored the SOD, CAT activities and GSH level in the hepatic tissue. These results suggested that MSH attenuated ethanol-induced oxidative stress via restoring the compromised capacity of the liver antioxidant system.

Nrf2 is a transcriptional factor that regulates the expression of antioxidant and phase II detoxification genes. As with other liver diseases mediated by oxidative stress, the protective role of Nrf2 in ALD has also been highlighted [53,54]. The hepatocyte-specific knockout of Kelch-like ECH-associated protein 1 (Keap1) in mice, which induces the activation of Nrf2, offered protection against liver injury by ethanol [55]. On the other hand, Nrf2-deficient mice showed susceptibility to ethanol exposure, exhibiting GSH depletion and increased mortality [56]. Research has reported that the total extract or lignans of *S. chinensis* exerted a hepatoprotective effect on the ethanol-induced liver injury via increasing the stability and transcriptional activity of Nrf2 [32,57]. Moreover, HSCF treatment increased the transcriptional activity of Nrf2 and the expression of its target genes in HepG2 cells [58]. And HSCF administration also increased the relative expression of Nrf2 mRNA levels in ethanol-intoxicated mice [12]. In the current study, MSH administration increased the relative mRNA expression of Nrf2, suggesting that the hepatoprotective effects of MSH against ethanol-induced oxidative stress are mediated by the modulation of the antioxidant system via Nrf2 activation.

Research has been conducted about the composition and preventive effect of SF and HSCF. The main active chemical constituents of *S. chinensis* include lignans and polysaccharides [59]. Gomisin N, one of the lignans in SF, alleviated ethanol-induced hepatic steatosis, lipogenesis, and inflammation in mice [19]. The polysaccharide fraction of *S. chinensis* exerted the protective effect on ethanol-induced oxidative stress via reducing CYP2E1 expression and elevating SOD activity [10]. As for *H. dulcis*, several flavonoids such as myricetin, dihydromyricetin, quercetin, gallocatechin and polysaccharides have been identified as active components in its seeds, fruits, and peduncles [15]. Specifically, inhibitory effects of myricetin on the fatty acid biosynthesis in ethanol-fed mice have been reported [60]. And the hepatoprotective effect of polysaccharides isolated from peduncles of *H. dulcis* have also been reported [61].

The chemical composition of herbs and their extracts can be influenced by various factors, including the timing of harvest and the method of extraction. Furthermore, the specific parts or species of plants used may vary depending on traditional medicine practices or regional customs. Such variations could affect the biological effectiveness of herbs, highlighting the importance of carefully controlling these factors to achieve reproducible experimental outcomes. In this research, the fruit of *S. chinensis* and the peduncle of *H. dulcis* were utilized to manufacture each hot water extract. The manufacturing processes of fSFP, HSCF, and MSH were documented for standardization and quality control. Additionally, the indicative constituents of both herbs, schizandrin and myricetin, in MSH were identified using HPLC analysis (Figures S1 and S2). We expect that these findings will improve the reliability of the efficacy and reproducibility of future studies. Nevertheless, the current study did not provide a specific identification of the constituent components within MSH that exhibited synergistic protective effects. Therefore, additional studies on the pharmacological interactions, bioavailability, and content of active components that are responsible for MSH’s synergistic effect are needed.

Over the years, preclinical experimental models of ALD have been continuously developed for the comprehensive understanding of its pathogenesis. The binge-feeding method, in which ethanol is administered acutely through oral gavage, is widely used due to its simplicity in replicating liver damage caused by excessive alcohol consumption. The chronic feeding method, providing animals with free access to ethanol through drinking water for an extended period of times, mimics chronic alcohol consumption [62]. Moreover, chronic plus binge feeding has been developed for reflecting a drinking pattern of alcoholic hepatitis patients [63]. In the present study, we adopted subacute ethanol-mediated liver injury using 14 days of binge feeding with simultaneous MSH administration to investigate its protective effects. However, the current model is known to allow the observation of early phenomena such as liver cell death and steatosis in the pathogenesis of ALD, while severe progression such as fibrosis or mortality is not observed. Therefore, to validate the efficacy of MSH on a broad spectrum of ALD, it would be necessary to conduct additional research using chronic and severe experimental models such as chronic plus binge feeding. Furthermore, exploring the therapeutic effects of post-treatment with MSH after the occurrence of hepatic damage and steatosis by ethanol administration will provide a more profound understanding of MSH’s effect in treating ALD.

## 5. Conclusions

The current investigation found that 50, 100, and 200 mg/kg of HSCF administration significantly alleviated the ethanol-induced liver toxicity. MSH lowered lipid accumulation in liver via modulating the expression of genes involved in fatty acid synthesis and oxidation. Moreover, MSH exerted an anti-oxidative property through enhancing the antioxidant defense system capacity by Nrf2 activation. Furthermore, the significantly greater effects of MSH in anti-steatosis, anti-oxidative stress, and anti-inflammation, compared to fSFP or HSCF single administration, indicate the synergistic hepatoprotective effect against ethanol-induced liver toxicity.

## Figures and Tables

**Table 1:** Information of oligonucleotides used in RT-qPCR.

Target Gene	Primer Sequence (Forward, Reverse)	Gene ID	Product Size
*SREBP-1c*	5′-GATGTGCGAACTGGACACAG-3′, 5′-CATAGGGGGCGTCAAACAG-3′	20787	104 bp
*SCD1*	5′-CCCCTGCGGATCTTCCTTAT-3′, 5′-AGGGTCGGCGTGTGTTTCT-3′	20249	114 bp
*ACC1*	5′-GCCATTGGTATTGGGGCTTAC-3′, 5′-CCCGACCAAGGACTTTGTTG-3′	107476	112 bp
*FAS*	5′-GCTGCGGAAACTTCAGGAAAT-3′, 5′-AGAGACGTGTCACTCCTGGACTT-3′	14104	84 bp
*PPARγ*	5′-AGTGGAGACCGCCCAGG-3′, 5′-GCAGCAGGTTGTCTTGGATGT-3′	19016	64 bp
*DGAT2*	5′-AGTGGCAATGCTATCATCATCGT-3′, 5′-AAGGAATAAGTGGGAACCAGATCA-3′	67800	149 bp
*PPARα*	5′-ATGCCAGTACTGCCGTTTTC-3′, 5′-GGCCTTGACCTTGTTCATGT-3′	19013	220 bp
*ACO*	5′-GCCCAACTGTGACTTCCATT-3′, 5′-GGCATGTAACCCGTAGCACT-3′	74121	113 bp
*CPT1*	5′-GCACTGCAGCTCGCACATTACAA-3′, 5′-CTCAGACAGTACCTCCTTCAGGAAA-3′	12894	324 bp
*Nrf2*	5′-CGAGATATACGCAGGAGAGGTAAGA-3′, 5′-GCTCGACAATGTTCTCCAGCTT-3′	18024	79 bp
*β-actin*	5′-CTGTCGAGTCGCGTCCA CCCGCGAG-3′, 5′-CTCGCGGGTGGACGCGACTCGACAG-3′	11461	516 bp

RT-qPCR, Quantitative reverse transcription polymerase chain reaction; SREBP-1c, Sterol regulatory element-binding protein-1c; SCD1, Stearoyl-CoA desaturase 1; ACC1, Acetyl-CoA carboxylase 1; FAS, Fatty acid synthase; PPAR, Peroxisome proliferator-activated receptor; DGAT2, Diacylglycerol acyltransferase 2; ACO, Acyl-CoA oxidase; CPT1, Carnitine palmitoyltransferase 1; Nrf2, Nuclear factor erythroid 2-related factor-2.

## Data Availability

Not applicable.

## Supplementary Materials

The following supporting information can be downloaded at: https://www.mdpi.com/article/10.3390/antiox12081602/s1, Figure S1: Flow chart for manufacturing hot water extracts. Figure S2: Identification of MSH using high-performance liquid chromatography (HPLC) analysis. Table S1: Instrument condition of HPLC analysis.
