# Supplementary material for: Synergistic Protective Effect of Fermented Schizandrae Fructus Pomace and Hoveniae Semen cum Fructus Extracts Mixture in the Ethanol-Induced Hepatotoxicity
Source: Antioxidants (Basel). 2023 Aug 11;12(8):1602. doi: 10.3390/antiox12081602 (PMC10451898; doi:10.3390/antiox12081602)
Supplement: Supplementary file 1 [file antioxidants-12-01602-s001.zip › antioxidants-2546345-supplementary.pdf]

antioxidants

MDPI

Supplementary Information

Synergistic Protective Effect of Fermented Schizandrae Fructus Pomace and Hoveniae Semen cum Fructus Extracts Mixture in the Ethanol-Induced Hepatotoxicity

Kyung-Hwan Jegal [1], Hye-Rim Park [2,3], Beom-Rak Choi [3], Jae-Kwang Kim [4,*] and Sae-Kwang Ku [2,*]

1 Department of Korean Medical Classics, College of Korean Medicine, Daegu Haany University, Gyeongsan 38610, Republic of Korea; jegalkh@dhu.ac.kr
2 Department of Anatomy and Histology, College of Korean Medicine, Daegu Haany University, Gyeongsan 38610, Republic of Korea; hrpark@nutracore.co.kr
3 Nutracore Co., Ltd., Suwon 16514, Republic of Korea; brchoi@nutracore.co.kr
4 Department of Physiology, College of Korean Medicine, Daegu Haany University, Gyeongsan 38610, Republic of Korea
* Correspondence: kim-jk@dhu.ac.kr (J.-K.K.); gucci200@hanmail.net (S.-K.K.)

S1. Manufacturing process of fermented Schizandrae Fructus pomace (fSFP) and Hoveniae Semen cum Fructus (HSCF) hot water extracts.

fSFP, HSCF, and 1:1 mixture of fSFP and HSCF (MSH) extracts were supplied from Nutracore (Suwon, Korea). Briefly, 100 kg of each raw material (fSFP and HSCF) was extracted with hot water (800 L) for 8 h, and then filtered. Second extraction with hot water (500 L, 4–5 h) was conducted, and filtered once more. Finally, the resulting extracts were concentrated using evaporator, and dried using a spray drier (Figure S1). Mixed formulas (MSH) were prepared by dissolving same amount of fSFP and HSCF in the distilled water.

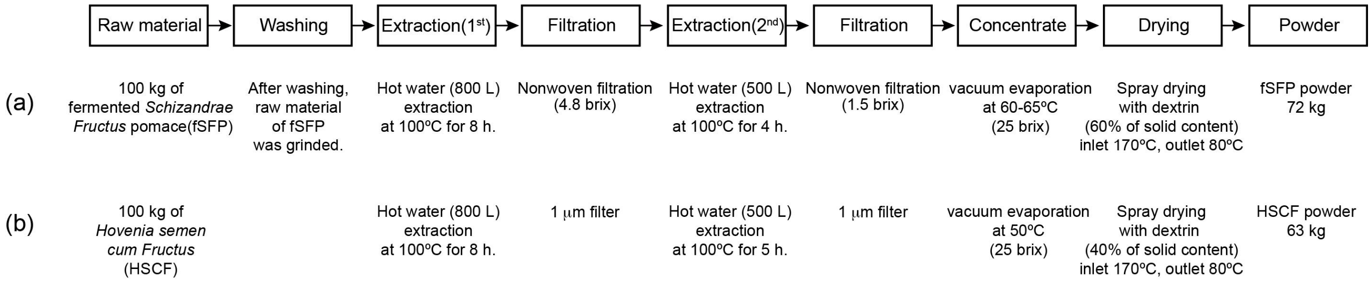

Figure S1. Flow chart for manufacturing hot water extracts. **(a)** Manufacturing process of fermented Schizandrae Fructus pomace (fSFP) and **(b)** Hoveniae Semen scum Fructus (HSCF).

S2. Identification of marker compounds in MSH using high-performance liquid chromatographic (HPLC) analysis

For standardization and quality control of raw material, schizandrin and myricetin content in MSH were identified using HPLC. Schizandrin and myricetin are indicative compounds of SF and HSCF, respectively. Schizandrin standard compound was purchased from Sigma-Aldrich (St. Louis, MO, USA). And myricetin standard compound was obtained from ChromaDex (Irvine, CA, USA).

To detect schizandrin in MSH, 200 mg of MSH was dissolved in distilled water to make 20 mL of solution, and subjected to ultrasonic extraction for 30 min. After filtration, the resulting sample was used for analysis. 2 mg of schizandrin standard was dissolved in water to make 20 mL of solution. From this, a standard solution was prepared by

diluting 1 mL of standard stock solution with distilled water to a final volume of 10 mL. 10 μL of the sample and standard solution were for HPLC analysis under conditions described in Table S1. Briefly, HPLC analysis was performed using Agilent HPLC system (Agilent, Waldbronn, Germany) equipped with C18 column (CAPCELL PAK C_{18}, 4.6 × 250 mm, 5 μm) at 30°C column temperature. The mobile phase consisted of water with 0.05% TFA (A) and acetonitrile (B). HPLC gradient conditions were follows: 0 min (A 55%, B 45%), 40 min (A 55%, B 45%), 41–55 min (A 5%, B 95%) with the 0.7 mL/min flow rate. Absorbance at 254 nm wavelength was detected.

To detect myricetin in MSH, 1 g of MSH was dispersed in 10 mL of distilled water and then mixed with 40 mL of methanol to make 50 mL of solution. After ultrasonic extraction for 30 min and filtration, the resulting sample was used for analysis. For the preparation of the myricetin standard solution, 1.0 mg of myricetin standard was dissolved in 80% methanol to make 80 mL of solution. 20 μL of the sample and standard solution were for HPLC analysis under conditions described in Table S1. Briefly, HPLC analysis was performed using HPLC system (Agilent) equipped with C18 column (Eclipse Plus C_{18}, 4.6 × 250 mm, 5 μm) at 30°C column temperature. HPLC gradient conditions were follows: 0 min (A 80%, B 20%), 25 min (A 80%, B 20%), 26 min (A 5%, B 95%), 35 min (A 5%, B 95%) with the 1.0 mL/min flow rate. Absorbance at 372 nm wavelength was detected.

Table S1. Instrument condition of HPLC analysis

**Parameter**	**Schizandrin**	**Myricetin**
Chromatography	Agilent 1200-DAD	Agilent 1100-DAD
Detector	UV (254 nm)	UV (372 nm)
Column	CAPCELL PAK C_{18} (4.6 × 250 mm, 5 μm)	Eclipse Plus C_{18} (4.6 × 250 mm, 5 μm)
Mobile phase	A: water with 0.05% of TFA<br>B: acetonitrile	A: water with 0.05% of TFA<br>B: acetonitrile
Flow rate	0.7 mL/min	1.0 mL/min
Injection vol.	10 μL	20 μL
Column temp.	30°C	30°C

HPLC analysis showed that one peak of MSH extract solution in water matched with schizandrin at retention time of approximately 20.578 min (Figure S2a). In addition, one peak of MSH extract solution in 80% methanol matched with myricetin at retention time of approximately 17.489 min (Figure S2b). Quantification based on their peak areas and retention times revealed that MSH contains 0.6 mg/g of schizandrin and 0.17 mg/g of myricetin.

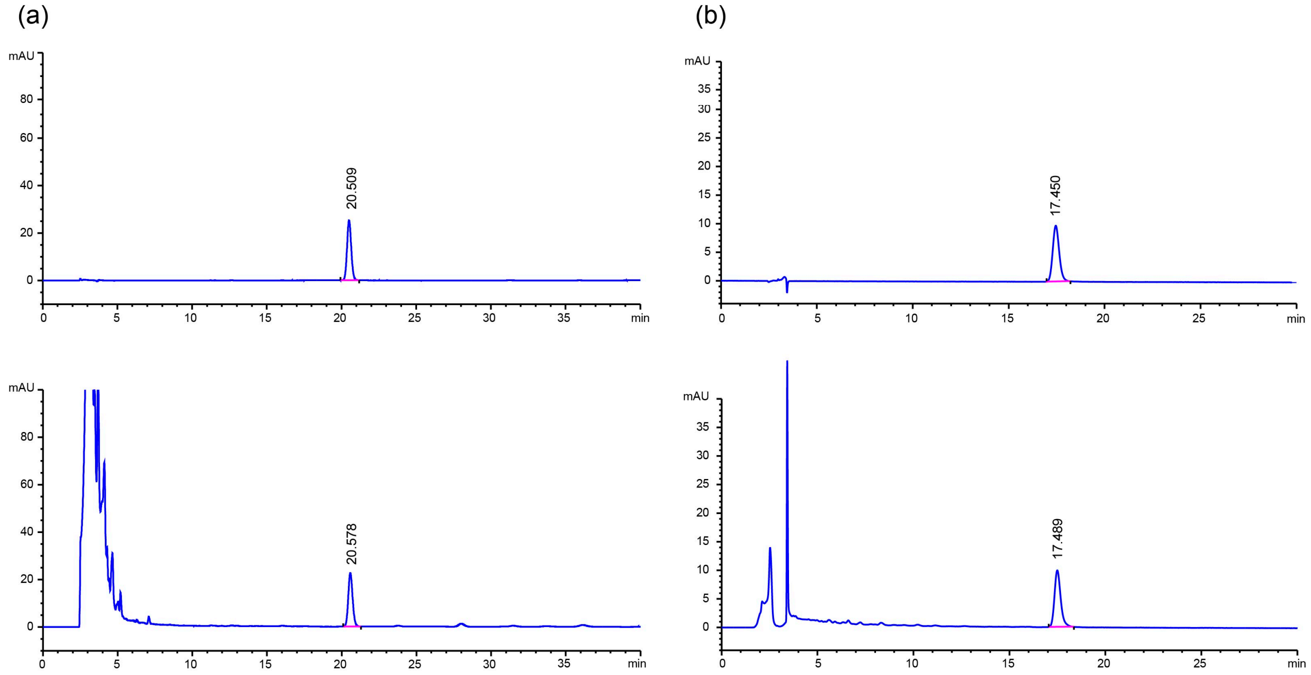

Figure S2. Identification of MSH using high performance liquid chromatography (HPLC) analysis. (a) Chromatogram of schizandrin standard (upper) and MSH (lower). (b) Chromatogram of myricetin standard (upper) and MSH (lower).
